# CaDAVEr: a metagenome-assembled genome catalog of microbial decomposers across vertebrate environments

**DOI:** 10.1128/mra.01323-24

**Published:** 2025-09-23

**Authors:** Valerie A. Seitz, Bridget B. McGivern, Michael Shaffer, Mikayla A. Borton, Aeriel D. Belk, Parsa Ghadermazi, Cameron Martino, Liat Shenhav, Anru R. Zhang, Pixu Shi, Alexandra Emmons, Heather Deel, Zhenjiang Zech Xu, Victoria Nieciecki, Qiyun Zhu, Kalen Cantrell, Asa Ben-Hur, Sasha C. Reed, Greg C. Humphry, Gail Ackermann, Daniel McDonald, Siu Hung Joshua Chan, Melissa Connor, Derek A. Boyd, Jake Smith, Jenna M. S. Watson, Giovanna Vidoli, Dawnie Steadman, Aaron M. Lynne, Sibyl Bucheli, David O. Carter, Zachary M. Burcham, Rob Knight, Kelly C. Wrighton, Jessica L. Metcalf

**Affiliations:** 1 Department of Animal Sciences, Colorado State University 728642, Fort Collins, Colorado, USA; 2 Department of Soil and Crop Sciences, Colorado State University 124498 , Fort Collins, Colorado, USA; 3 Department of Chemical and Biological Engineering, Colorado State University 3447, Fort Collins, Colorado, USA; 4 Department of Pediatrics, University of California San Diego 547075, La Jolla, California, USA; 5 Center for Studies in Physics and Biology, The Rockefeller University 371261 , New York, New York, USA; 6 Institute for Systems Genetics, New York Grossman School of Medicine, New York University 5894, New York, New York, USA; 7 Department of Computer Science, New York University 171561, New York, New York, USA; 8 Departments of Biostatistics and Bioinformatics and Computer Science, Duke University 3065, Durham, North Carolina, USA; 9 Department of Computer Science, Duke University 327138, Durham, North Carolina, USA; 10 School of Food Science and Technology, Nanchang University 621983, Nanchang, Jiangsu, China; 11 Graduate Program in Cell and Molecular Biology, Colorado State University 3447, Fort Collins, Colorado, USA; 12 School of Life Sciences, Arizona State University 69991, Tempe, Arizona, USA; 13 Center for Fundamental and Applied Microbiomics, Arizona State University 7864, Tempe, Arizona, USA; 14 Department of Computer Science and Engineering, UC San Diego 214553, La Jolla, California, USA; 15 Department of Computer Science, Colorado State University 3447, Fort Collins, Colorado, USA; 16 U.S. Geological Survey, Southwest Biological Science Center 302052, Moab, Utah, USA; 17 Forensic Investigation Research Station, Colorado Mesa University 3580, Grand Junction, Colorado, USA; 18 Forensic Anthropology Center, Department of Anthropology, University of Tennessee 36542, Knoxville, Tennessee, USA; 19 Department of Social, Cultural, and Justice Studies, University of Tennessee at Chattanooga 14733, Chattanooga, Tennessee, USA; 20 Mid-America College of Funeral Service 32456 , Jeffersonville, Indiana, USA; 21 Department of Biological Sciences, Sam Houston State University 171821, Huntsville, Texas, USA; 22 Laboratory of Forensic Taphonomy, Forensic Sciences Unit, School of Natural Sciences and Mathematics, Chaminade University of Honolulu 621655, Honolulu, Hawaii, USA; 23 Center for Microbiome Innovation, University of California San Diego 8784, La Jolla, California, USA; 24 Department of Bioengineering, University of California San Diego 8784, La Jolla, California, USA; Montana State University, Bozeman, Montana, USA

**Keywords:** metagenomics, necrobiome, MAG, forensics, decomposition, vertebrates

## Abstract

Microbial degradation of organic matter is a fundamental Earth process, yet a mechanistic understanding of microbial metabolisms and successional ecology involved in decomposition remains poorly understood. Here, we announce the recovery of 277 cadaver-associated soil metagenome-assembled genomes to enhance our understanding of vertebrate decomposition microbial processes.

## ANNOUNCEMENT

Decomposition of organic material, including plants, animals, and their byproducts, is one of the most important ecosystem processes on Earth, requiring complex metabolisms and multi-trophic food webs to efficiently recycle nutrients. Animal tissue decomposition is particularly understudied despite its potential role for innovating agricultural and forensic tools. Microbial communities critical to animal tissue decomposition, also known as the necrobiome (1), lack metagenomic data characterizing this metabolism. Using soils longitudinally collected from 36 terrestrially decomposing human cadavers (*n* = 21 timepoints per donor) across three U.S. locations (Knoxville, TN; Grand Junction, CO; Huntsville, TX), we present a genomic resource for microbial decomposers of animal tissue (2). Here, we introduce 277 medium- and high-quality metagenome-assembled genomes (MAGs) comprising the CaDAVEr (Catalog of microbial Decomposers Across Vertebrate Environments) database. We highlight 29 taxa previously identified as central members to a co-occurrence network associated with advanced decomposition (Table 1) (2). We have previously shown that these microbes are ubiquitous vertebrate decomposers with insects as a likely vector (2). CaDAVEr members are largely undetected in large databases of host-associated or soil microbial communities, supporting the notion that the necrobiome has an untapped suite of microbial community and functional diversity (2).

**TABLE 1:** Quality and genome information of twenty-nine decomposer metagenome-assemble genomes recovered from vertebrate decomposition soils

MAG ID	NCBI accession	Genome size (bp)	Coverage (×)	Number of scaffolds	N50	Completeness (%)	Contamination (%)	GC	# Predicted genes	Taxonomy (genus)
CMU_advanced.final.11	SAMN35122743	1,738,034	111	186	12,259	89.67	0.88	44.3	1,802	g__Wohlfahrtiimonas
CMU.bins.224	SAMN35122767	1,447,706	78	42	84,618	81.62	0.47	29.9	1,462	g__JASLAT01
CMU.bins.248	SAMN35122772	1,960,261	382	271	8,493	80.27	2.29	40.5	1,759	g__Ignatzschineria
CMU.bins.298	SAMN45170734	1,379,843	3.71502592	260	5,412	71.93	2.18	41.6	1,309	g__Ignatzschineria
CMU.bins.69	SAMN45170740	2,179,327	13.2268609	266	10,819	79.66	6.9	41.2	1,933	g__Ignatzschineria
SHSU_active.final.55	SAMN35122805	2,230,926	31	222	16,150	95.52	0.94	37.6	2,311	g__Savagea
SHSU_active.final.81	SAMN35122806	2,756,840	55	165	23,680	97.42	0	33.7	2,234	g__Bacteroides_E
SHSU_advanced.final.156	SAMN35122812	1,997,474	7	274	9,700	90.58	2.08	37.7	1,899	g__Bacteroides_E
SHSU_advanced.final.35	SAMN35122823	1,258,874	23	248	5,499	74.04	1.14	38.4	1,390	g__Vagococcus_A
SHSU.bins.123	SAMN35122833	3,542,934	63	533	7,822	82.18	4.29	51	3,625	g__Morganella
SHSU.bins.132	SAMN35122835	2,116,720	54	106	25,442	99.71	1.17	45.6	1,961	g__CALZBJ01
SHSU.bins.167	SAMN35122846	2,263,551	21	281	10,811	88.41	1.95	33.6	2,071	g__Bacteroides_E
SHSU.bins.175	SAMN35122849	1,981,783	25	263	9,902	78.09	6.73	41.9	1,749	g__Ignatzschineria
SHSU.bins.195	SAMN35122857	2,596,507	37	254	15,705	88.91	1.17	39.7	2,325	g__Ignatzschineria
SHSU.bins.354	SAMN35122895	1,538,193	46	131	14,935	94.25	0.58	45.4	1,533	g__JAAZCI01
SHSU.bins.365	SAMN35122898	1,752,486	84	45	63,825	97.27	0	44.1	1,629	g__Ignatzschineria
SHSU.bins.50	SAMN35122928	4,056,206	45	729	6,174	51.88	0	58	4,297	g__Klebsiella
SHSU.bins.517	SAMN35122931	1,912,837	24	134	22,260	87.43	3.8	37	1,898	g__Wohlfahrtiimonas
SHSU.bins.531	SAMN35122934	1,451,374	171	291	5,212	62.9	3.31	40.5	1,361	g__Ignatzschineria
SHSU.bins.89	SAMN35122953	1,507,431	46	222	8,450	60.92	4.78	38.7	1,408	g__Ignatzschineria
UTK_active.final.18	SAMN35122955	1,605,292	242	244	7,889	77.55	0.7	47.6	1,679	g__Thiopseudomonas
UTK_active.final.26	SAMN35122956	1,609,873	46	254	7,156	85.83	4.17	46.1	1,799	g__Savagea
UTK.bins.107	SAMN35122961	2,578,716	47	455	6,343	79.81	1.15	40.1	2,608	g__Acinetobacter
UTK.bins.126	SAMN35122964	1,906,363	15	352	5,909	71.72	9.56	40.4	1,925	g__Ignatzschineria
UTK.bins.19	SAMN35122978	2,219,372	43	159	23,955	95.47	1.3	39.5	1,957	g__Bacteroides_E
UTK.bins.42	SAMN35122984	1,863,275	18	142	19,613	93.6	0.39	40	1,884	g__Savagea
UTK.bins.50	SAMN35122986	1,565,609	48	53	63,371	96.34	0.91	36.5	1,510	g__CALAZW01
UTK.bins.71	SAMN35122994	1,322,987	34	287	4,565	64.9	5.35	46	1,491	g__Savagea
UTK.bins.74	SAMN45170746	1,649,164	11.0139592	25	146,272	97.95	0	44.3	1,534	g__JAAZCI01

Swab samples were collected across decomposition stages from soils adjacent to 36 human cadavers (*n* = 756). Soil controls (*n*  =  9), blank controls (*n*  =  102), and no-template PCR controls (*n*  =  15) were included. DNA was extracted using the PowerSoil DNA isolation kit 96-htp (MoBio Laboratories) and prepared for shallow metagenomic sequencing. Sequencing (2 × 151 bp) was completed on either an Illumina HiSeq 4000 or an Illumina NovaSeq 6000, resulting in 1,271 Gbp of sequencing and 4,211,654,692 total reads. Adapters were removed, and reads were filtered using Atropos (*q* = 15, --minimum-length 100, v.1.1.24) (3). Bowtie2 (v.2.2.3) (4) was used to align human sequences against the Genome Reference Consortium Human Build 38 patch release 13, removing all data that matched the reference. Resulting filtered SAM files were converted to FASTQ format with samtools (5) (v.1.3.1) and bedtools (6) (v.2.26.0). Samples with <500 k reads were removed from the analysis as a quality control measure, resulting in a final metagenomics data set of 569 hip-adjacent soil samples, five soil controls, and four no-template controls (*N* = 575).

**Fig 1:**
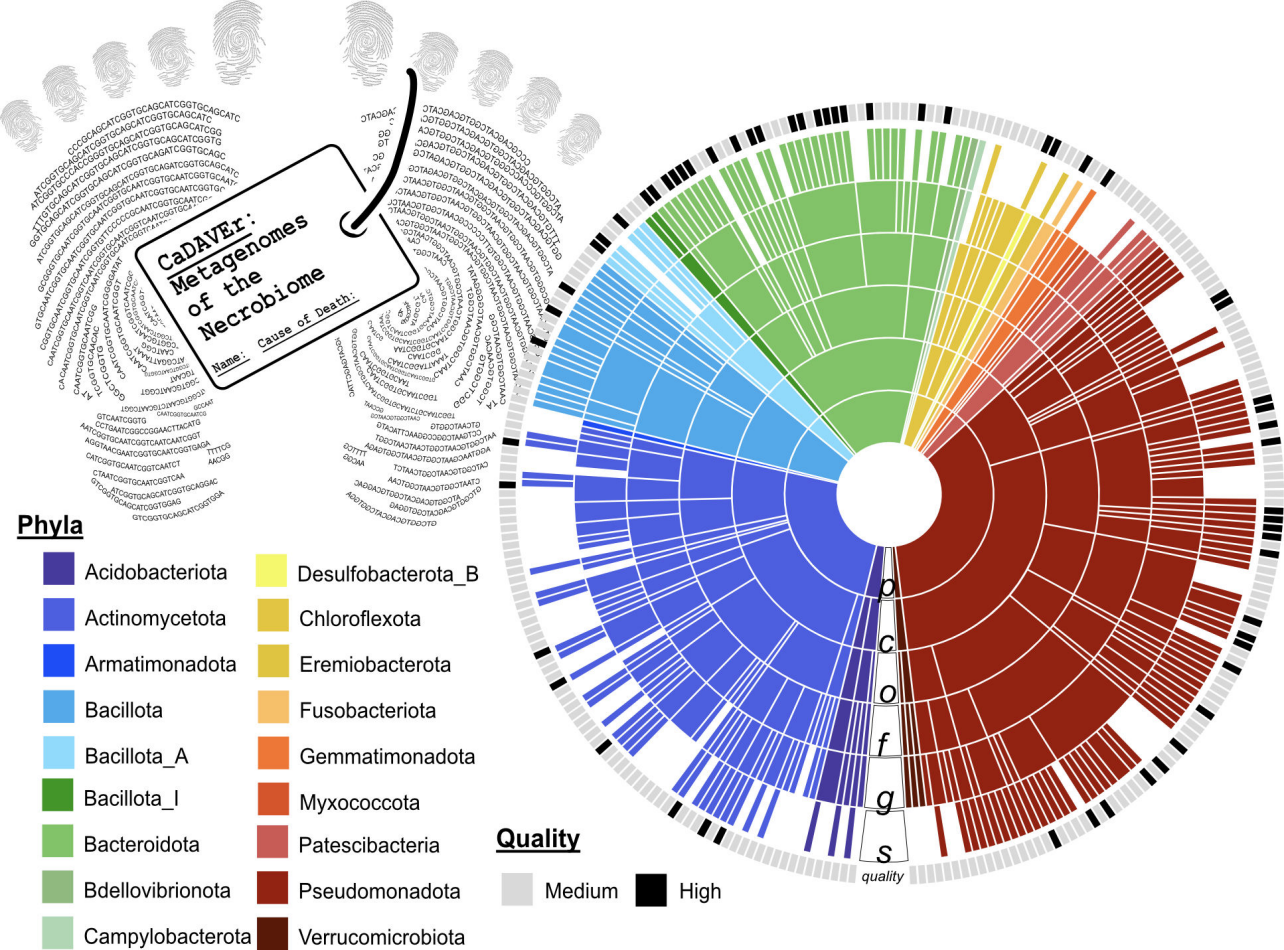
CaDAVEr yields a robust MAG database of microbial decomposers. The taxonomy of the 277 dereplicated MAGs is shown by sequentially colored rings ordered from phylum (P), class (C), order (O), family (F), genus (G), to species (S) assignment. Ring color corresponds to phyla, with the taxonomic assignment denoted in the legend to the left. Gaps at each level represent MAGs that were unclassified at that level of taxonomy (according to GTDB-tk v.2.4.0, r220). The outer ring shows the MAG quality classification, where gray is a medium-quality MAG (completion >50% and contamination <10%), and black is a high-quality MAG (completion >90% and contamination <10%).

Using protocols described previously to maximize genome recovery (7, 8), metagenomes were assembled by geographic site and decomposition stage (MEGAHIT, v.1.2.9, –k-min 41) (9) with assembled scaffolds >2,500 kb binned into MAGs (MetaBAT2, v.2.12.1) (10). Bin quality was assessed by checkM (v.1.1.2) (11), retaining only medium- and high-quality MAGs, which resulted in 1,130 total MAGs (>50% estimated completion and <10% contamination). MAGs were then dereplicated at 99% identity (dRep, v.2.6.2) (12), resulting in 277 MAGs in CaDAVEr, of which 24% are considered high quality (Fig. 1). MAGs were assigned taxonomy via GTDB-tk (v.2.4.0, r220) (13), and genes were annotated using DRAM (v.1.0.0) (14) and were predominantly assigned to the phyla *Pseudomonadota* (dark red, *n* = 99), *Actinomycetota* (dark blue, *n* = 70), and *Bacteroidota* (light green, *n* = 41; Fig. 1). The package default parameters were used unless otherwise specified. Resources of the CaDAVEr database broaden the taxonomic and functional characterization of the vertebrate necrobiome, contributing important phylogenetic and genomic information within decomposition.

## Data Availability

Raw metagenomic sequencing data and sample metadata are available on Qiita (study 14989) and ENA accession PRJEB62460 (ERP147550). The 277 dereplicated MAGs and their taxonomy can be found publicly on Zenodo (https://zenodo.org/records/7938240) and NCBI under project PRJNA973116. The full table detailing the MAG quality and accession numbers for all 277 MAGs can be found on Zenodo (https://zenodo.org/records/15660361).
